# A machine learning study highlighting the challenges of fidgety movement recognition using vision and inertial sensors

**DOI:** 10.1038/s41598-025-28523-3

**Published:** 2026-01-05

**Authors:** Falco Lentzsch, Frédéric Li, Friederike Pagel, Margot Lau, Andrea Kock, Hanna Marie Röhling, Anne Stein, Maciej Baranowski, Marco Maass, Hannes Hölzl, Sebastian Glende, Sebastian Mansow-Model, Ute Thyen, Marcin Grzegorzek

**Affiliations:** 1 German Research Center for Artificial Intelligence (DFKI), Luebeck, 23562 Germany; 2 Sozialpädiatrisches Zentrum (SPZ), UKSH, Luebeck, 23562 Germany; 3 Motognosis GmbH, Berlin, 10119 Germany; 4 YOUSE GmbH, Berlin, 13187 Germany; 5 University of Luebeck, Luebeck, 23562 Germany; 6 University of Economics of Katowice, Katowice, 40-287 Poland

**Keywords:** General movement assessment, Body tracking, Inertial measurement units, Deep learning, Feature disentanglement, Paediatric research, Neurological disorders, Machine learning

## Abstract

Past medical research has shown that infantile movement and early neurological development are closely linked. Fidgety Movements that are reflex-like movement occurring in healthy infants less than 20-week of age have proven to be especially important, as past studies have highlighted that their absence is strongly correlated with the future development of neurological disorders like Cerebral Palsy. To provide a timely intervention, the General Movement Assessment was proposed as a screening medical procedure carried out by clinical personnel specifically trained to recognize Fidgety Movements. Because of its high cost in time and resources, several initiatives to automatize General Movement Assessment using machine learning techniques have been proposed in the literature. However none has managed to emerge as state-of-the-art so far. To investigate this problem, we conducted a study using deep learning approaches to learn disentangled feature representations for the recognition of Fidgety Movements using RGB-D video and Inertial Measurement Unit data acquired from 95 infants (average age: $13.79 \pm 1.40$ weeks). Our results show that while it is possible to learn features that characterize movement independently of subject information, obtaining feature representations that consistently generalize to subjects unseen during training remains challenging. More specifically, we observe that both the vision- and sensor-based modalities have specific challenges to be addressed for the recognition of Fidgety Movements. We discuss them and provide recommendations to help researchers interested in investigating this problem in the future.

## Introduction

The three first years after birth are the most critical period of time in the life of an individual regarding human brain development^1^. During that time, the majority of the neural connections are formed and the brain doubles in size, with the neurological development finally completing in early adulthood. It is therefore extremely important to closely monitor infants during this period of their life to ensure that no abnormalities with potentially lifelong lasting consequences occur. Neurological development has been shown to strongly correlate with infant gestures and movements. This relationship was first described in the 90 s by Heinz Prechtl^2^, who developed the so-called General Movement Assessment (GMA)^3^ which focuses on analyzing General Movements (GMs). GMs are spontaneous reflex-like movements observable from birth until around six months of age. During this period, GMs undergo changes in amplitude, speed, and acceleration, comprising most parts of the body (e.g., neck, limbs). Depending on the stage of development of the child, GMs are further classified into preterm GMs, which typically occur between 28 and 38 gestational weeks, and term or fidgeting movements, which are usually observed between 38 gestational weeks and 12 weeks post-term. Subsequently, Fidgety Movements (FMs) occur between 10 and 20 weeks post-term^4^. These different time windows correspond to different phases of the infant’s neuromotor maturation, and play a crucial role in the early detection of possible neurological abnormalities.

From a clinical perspective, one of the most important categories are FMs^5^. FMs are multidirectional movements characterized by small amplitude, moderate speed, and variable acceleration. Occasionally they can be observed in isolated parts of the body. However, they often occur with varying frequencies and amplitude in different body parts. They often do not appear at the same time but progress through the child’s body^6^. In addition, these movements are often superimposed on the top of larger motor movements, such as tightening the legs or raising the arm, making the detection of FMs considerably more difficult^6^. They also constitute the majority of observed movements at this stage of infant development. The FMs of a child can be classified in five different categories characterizing the quantity of movement^7^: 1- Continual FMs (F++) that can occur in all body parts with a very short pause in-between them (approximately one to two seconds); 2- Intermittent FMs (F+) that are similar to continual ones, but with longer pauses in-between them (approximately 10 seconds); 3- Sporadic FMs (F+-) that occur only in some body parts, with in-between pauses ranging from three seconds to one minute. These type of FMs are commonly seen from six to eight weeks post term, and during the time when FMs fade out; 4- Abnormal FMs (AF) that are really rare and are mainly described as ”fidgety-like” movements, but differ in speed and amplitude. Some studies have also shown that these types of FMs are often diagnosed in infants with trisomy 21 or autism; 5- Absent FMs (F-) that designates the nearly complete absence of FMs.

These five aforementioned categories are observed during the GMA to assess only the quantity of FMs, but not their quality. GMA is the most sensitive and specific measure for early detection of Cerebral Palsy (CP) with a sensitivity of 98% and a specificity of 91% - outperforming cranial ultrasound, neurological examination and MRI - but does not predict disease severity^8^. Using a Gestalt approach, trained observers detect abnormal general movements (e.g., limited variation, cramped synchronized GM, absence of FMs) and ensure that the infant is awake and not crying. For a more detailed assessment of both the infant motor repertoire, the quantity and quality of movements can be scored between 6 (very poor) and 28 (optimal) using the Motor Optimality Score (MOS). The latter is a comprehensive GMA that assesses typical motor movements, including fidgeting movements^8^.

A revised version of the MOS scores was developed by Einspieler and Prechtl in 2019^9^, which was used as the basis for the study presented in this paper, and describes the current State Of The Art (SOTA) approach of the GMA. FM are the most important component here, accounting for nearly $50\%$ of all assessment points (12 out of 26 points). During screening, children are observed by specially trained clinicians for a short period of time, which presents several difficulties. Above all, the process is very time consuming, which leads to not all children undergoing this type of screening in a context of staff shortage. There are also major differences between clinicians in the way movements are assessed, which also makes it difficult to develop a standardized procedure that is easy to explain and transfer from one clinic to another.

A promising way to overcome those limitations is through the application of Machine learning (ML) to automate the entire process of recognizing FMs. Several approaches leveraging either visual or motion sensor data – most of the time independently – have been proposed in the past literature. Wearables and motion sensors have been increasingly used to detect activities in the past. Standard Red Green Blue (RGB) videos are the most commonly used modality in the past related work. A recent attempt to monitor the neurological development of infants was made by Morais et al.^10^, who investigated the problem of FM detection based on *OpenPose* body tracking in RGB videos. Several elaborate hand-crafted features characterizing limb and joints displacement and angles were computed from the *OpenPose* skeletons. ML models were then trained to output a score indicating the quality of FM movements, which was used to classify infants between normal or at risk. Two datasets S1 and S2 with 545 and 312 patients, respectively, were used for the study. The obtained evaluation performances remain however relatively mediocre for a binary classification problem, with an Area Under the Receiver Operating Characteristic Curve (AUROC) of $72.16\%$/$68.16\%$ on S1/S2, and a Specificity at $75\%$ sensitivity reaching only $54.11\%$/$44.80\%$on S1/S2. A second attempt by Morais et al.^11^ to bypass FM label scarcity was also performed. Hand-crafted features derived from the skeletons obtained by a fine-tuned version of *OpenPose* were computed to characterize movement direction, amplitude, speed and acceleration. A pseudo-labeling strategy consisting in training a random forest classifier on a labeled set, assigning labels to unlabeled samples with high classification confidence, and retraining the model was then implemented. The proposed approach was trained on two datasets of 1343 and 739 videos respectively. The obtained classification performances still remain low with an AUROC of $75.12\%$/$69.37\%$, and a specificity at $75\%$ sensitivity of $60.16\%$/$56.20\%$ on both respective datasets.

Approaches based on the processing of Inertial Measurement Unit (IMU) data are less commonly seen, but have also been attempted in the past. Gao et al.^12^ investigated the recognition of normal and abnormal movements using four IMUs placed at the wrists and ankles of the infants. A Multiple Instance Learning approach using low-dimensional Principle Component Analysis (PCA) features computed directly from the raw signals was implemented on a dataset of 34 infants, yielding an accuracy/precision/recall of $80\%$/$57\%$/$70\%$in a subject-dependent configuration (i.e. data from the same subjects in both training and testing sets). Waldheim et al.^13^ carried out a feasibility study to determine whether IMU placed on the legs, arms and chest of 12 infants could be used to determine abnormal body postures. Thresholds for features characterizing various angles between body parts were empirically determined to indicate whether a leg rise was occurring or not, as part of the MOS computation.

Finally, a few attempts trying to leverage both vision and motion sensor modalities can also be found. Rahmati et al.^14^ utilized RGB video data and six motion sensors placed on the infant’s wrists, ankles, head, and chest to classify between infants either healthy or at risk of CP. Manually crafted frequency-based features were extracted from both body tracks from the videos and the IMU data on a dataset of 78 infants. Classifiers were evaluated in a leave-one-sample-out subject-dependent setup, which yielded an accuracy/sensitivity/specificity of $91\%$/$86\%$/$92\%$ for the video features, and $85\%$/$88\%$/$71\%$for the IMU ones. Machireddy et al.^15^ explored a multimodal system combining video-based marker tracking with five IMUs placed on the legs, hands and chest of the infants to detect FMs. An extended Kalman filter was applied to estimate IMU acceleration, linear as well as angular velocity, magnetometer, and 3D position estimated from the video using projecting the color markers onto the image plane using camera calibration matrices. The estimated state parameters were then used as input of a Support Vector Machine (SVM) classifier trained for the classification of fidgety and non-fidgety movements on a dataset of 20 infants. An accuracy of $84\%$ was obtained in a subject-dependent setup.

Despite the numerous attempts to automate FM detection and the promising performances stated by some related work, a practical deployment of such solutions in a real-life clinical setting has yet to be reported. It can be noted that the most promising performances reported in the literature were obtained in subject-dependent setups. In 2019, Schmidt et al.^6^ also highlighted challenges in obtaining robust performances in a subject-independent setup for the classification of FM quality using RGB videos of the infants. Finally, investigations using alternative sensor modalities such as pressure sensing mats^16,17^ have also been undertaken, further hinting at the practical limitations of sensor and video-based modalities.

In this context, we carried out our own study aiming to develop a robust classification model for the recognition of FM using both video and inertial sensors on a dataset of 95 infants (49 males, 46 female, average age of $13.79 \pm 1.40$ weeks) from the University Hospital Schleswig-Holstein (UKSH) in Luebeck, Germany. We investigated ML approaches based on both feature engineering and deep feature learning. Models were trained on the acquired data using either video, inertial, or both modalities simultaneously to perform a binary classification approach between the classes “FM present” and “FM absent”, following the most common setup from related studies investigating FM detection^10,11,16,17^. To increase the cross-subject robustness of our models, we also evaluated methods based on feature disentanglement^18,19^ to learn a disentangled representation of the data that encodes movement information independently from any other personal characteristics, that we refer to as “subject identity” features. The results we obtained highlight the difficulty to get robust subject-independent classification performances for the automated recognition of FMs on large cohorts on infants, which aligns with the findings of past similar studies^10,11^. But discussions about the underwhelming classification performances in the past literature usually remain brief, with a focus on algorithmic shortcomings. For this reason, we investigate possible practical reasons that could explain why either the visual or inertial modality fails to provide features leading to a reliable detection of FM. We hope that the observations learned from our study will help researchers interested in automating the GMA with machine learning techniques avoiding similar obstacles.

In details, the contributions of our paper are as follows:

- We carry out a study investigating the classification of FM on a visual and inertial sensor dataset of 95 infants from the University Hospital Schleswig-Holstein (UKSH) in Luebeck, Germany. We train deep learning models taking either visual or inertial sensor modality data as input for the binary classification problem of “FM absent” against “FM present”, and report the results obtained in a subject-independent configuration.
- We apply feature disentanglement techniques to learn features characterizing both subject identity and movement in an independent manner, in an attempt to obtain features with better generalization capacity across subjects. We evaluate disentanglement visually, and with the help of a metric based on local neighborhood label entropy to characterize how the samples of all subjects are distributed in the feature space.
- We discuss in detail the obtained results, with an analysis focusing on explaining the shortcomings of both vision or inertial modalities from both a data quality and algorithmic perspective. Based on our observations, we provide practical recommendations to researchers interested in investigating the automation of the GMA using ML techniques to avoid the practical obstacles towards obtaining generalizable features that we faced.

This paper is organized as follows. In the *Materials and Methods* section, we describe the data collected for this study and the implemented methods. The *Experiments and Results* section presents the conducted experiments and the obtained results. In the *Discussion* section, we analyze the results and offer practical recommendations for researchers interested in automating the GMA. Finally, the *Conclusion* summarizes our key findings and contributions.

**Fig. 1:**
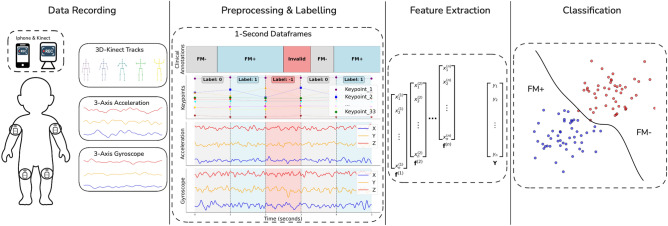
Visual illustration of the proposed machine learning framework for FM recognition.

## Material and methods

### Dataset

#### Data acquisition

**Fig. 2:**
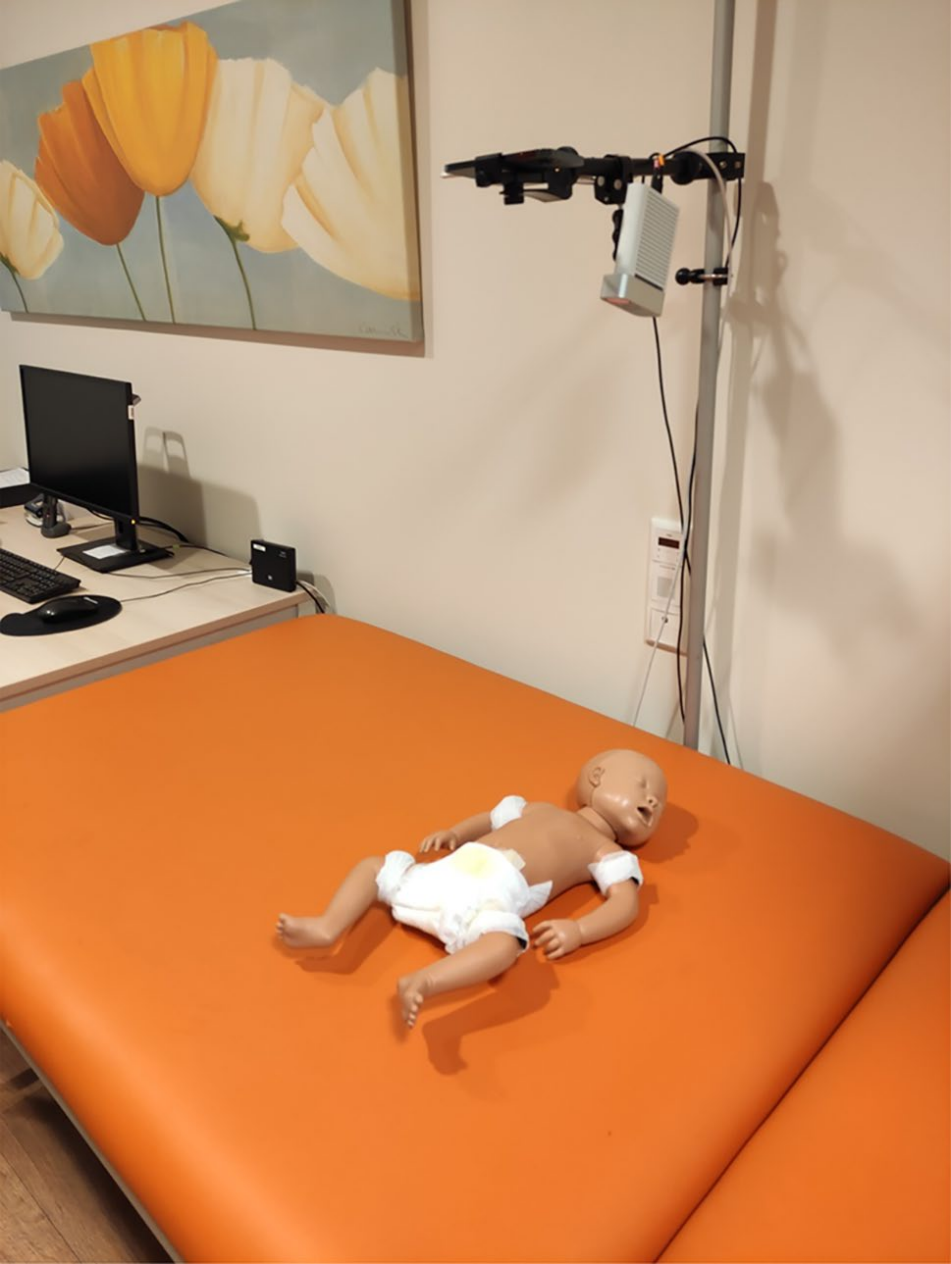
Data acquisition setup involving both visual and inertial sensors.

To carry out our study following the protocol shown in Fig. 1, a dataset of both Red Green Blue-Depth (RGB-D) and inertial data was acquired from 103 infants (52 males, 49 females, average age of $13.79 \pm 1.50$ weeks) in the University Hospital Schleswig-Holstein (UKSH) of Luebeck, Germany. The study was approved by the Ethics Committee of the University of Luebeck (application number: 21–319) and carried out in accordance with the Declaration of Helsinki regarding research involving human participants. For all participants of the study, an Informed Consent was signed by one of their legal guardians. Due to some technical issues during the data recording sessions, eight participants had to be excluded, leading to an effective dataset size of 95 infants (49 males, 46 females, average age of $13.79 \pm 1.40$ weeks). The average MOS is $21.69 \pm 4.92$, including six infants evaluated as high risk of abnormal neurological development by doctors from the UKSH. To ensure a consistent data collection from one infant to another, a controlled environment was setup in a room of the UKSH. The room was kept quiet, evenly illuminated with windows closed to minimize distractions, and warm to ensure the infants’ comfort. Infants were placed on a stretcher in supine position and dressed only in diapers to avoid occlusions during video analysis. The recording equipment consisted of two cameras and four IMUs. An iPhone 12 Pro captured high-resolution RGB video at 1080p and 60 frames per second, while a Microsoft Kinect Azure 3D camera (from now on referred to as Kinect) recorded at 1080p and 30 frames per second, providing both RGB and depth data. Both cameras were placed on a static wall-mounted support at a distance of approximately 90 cm above the infant. To minimize differences in the relative position of the infant to the cameras from one subject to another, the clinical experts who carried out the examination were instructed to place the infant in a standardized manner onto the stretcher, by centering them on the visual displays of both iPhone and Kinect cameras. Additionally, four MoveSense HR+ IMU (Movesense, Vantaa, Finland) recording three-axial acceleration and angular velocity at 200*Hz* were attached to the infants’ upper arms and thighs with medical gauze. To maintain consistency in the IMU placement across subjects, clinical doctors carrying out the GMA were instructed to place the sensors in the middle of the infants’ limbs. A sticker with an arrow was also placed on each IMU, with the instruction to point the arrow towards the head of the infant to standardize their orientation. The IMU data were transmitted via Bluetooth to the iPhone 12 Pro and synchronized with the iPhone RGB-D stream by an app developed by KAASA solution GmbH (Düsseldorf, Germany). An illustration of the data acquisition setup is provided in Fig. 2. The clinicians supervising the examination were instructed to record at least 300 seconds of data from each infant - a duration chosen to be significantly superior to the 30 to 60 seconds they usually require to assess FM in a regular examination session - so that enough FM data could be acquired. Some variance in the record durations could be observed depending on the mood of the infant, leading to an average duration of $447.62 \pm 112.77$ seconds, with a minimum of 137.54 seconds and maximum of 935.88 seconds.

#### Data annotation

To establish a ground truth for the recorded dataset, three clinical experts specifically trained in the detection and recognition of FM were asked to annotate the recorded iPhone videos using a web-based video annotation tool developed by KAASA solution GmbH. Using the annotation tool, each clinician had the option to mark the onset and offset of FM and to indicate the specific body regions where they were spotted. Annotating the data as “invalid”, i.e. unsuitable for FM evaluation (e.g. infant was in a bad mood, person interacting with the infant, etc.) was also provided as an option. The annotations were provided independently in a first stage with each expert annotating one third of the dataset. It should be noted that the comprehensive and continuous annotation of FM in time notably differs from both the traditional clinical procedure that usually requires the experts to briefly assess the presence and quality of FM in a few body parts, and the annotation strategy commonly used in related studies that only assign a binary label indicating the presence or absence of FM to segments of data^10,11,16,17^. For these reasons, subjectivity in the annotation strategies between different experts was observed (e.g. attention focused on different body parts, variability in the fine-graininess of the annotations), which led to notable differences in the provided annotations for some infants. In a second stage, the data of all infants was therefore again jointly annotated by at least two experts who used their initial annotations as starting basis, providing corrections and merging them as required.

#### Data preprocessing

To make the acquired data suitable for further analysis, the different sensor modalities were first synchronized. To synchronize both RGB-D Kinect and RGB iPhone streams, the infant-adapted version of the *OpenPose* body tracking algorithm^20^proposed by Chambers et al.^7^ was applied on both video streams. The *OpenPose* landmarks in the RGB frames were mapped into the corresponding depth frames of the videos and embedded in the 3D-point cloud of the infant body, providing 3D-landmark signals. In the following, these will be referred to as *DepthPose* landmarks. Temporal alignment was performed by determining the offset leading to the maximum correlation between time series of keypoint coordinates, followed by a visual confirmation carried out for each video.

The IMU data were processed to isolate motion data from each sensor. To ensure compatibility with other data sources, the six channels (three-axis acceleration and angular velocity) were resampled to a target frequency of 100*Hz* using linear interpolation to reduce the parameter complexity of the subsequent feature learning models. It was also decided to use the Kinect RGB-D videos in our study due to the extra information provided by the depth estimation. *DepthPose* was applied to extract the 3D coordinates of 33 body keypoints from each frame, leading to 99 time series of coordinates for each video.

Subsequently, the synchronized IMU and *DepthPose* data streams were segmented into one-second intervals using a non-overlapping sliding window approach. This resulted in data frames with dimensions $100 \times 6$ for each IMU sensor, and $30 \times 99$ for the *DepthPose* tracks. Labels corresponding to these segments were derived from the clinical annotations. More specifically, each segment was assigned to a single label among “fidgety” (FM+), “non-fidgety” (FM-) and “invalid” based on the majority annotation within the one-second window. To maintain label reliability, segments with ambiguous annotations (e.g. at the border between fidgety or non-fidgety) were excluded using a majority threshold of $60\%$, ensuring only clearly labeled data were included. Invalid segments were discarded from our study since they could contain either fidgety or non-fidgety movements, leading to a dataset of 32,220 one-second samples of synchronized IMU and Kinect data. It can be noted that even very fidgety infants with large MOS have periods of times without any FM, which makes the dataset relatively balanced between fidgety and non-fidgety periods despite the significantly higher number of healthy infants compared to those at risk. A total of 20,493 and 11,727 frames were respectively annotated as FM+ and FM-, leading to a class distribution of $63.60\%/36.40\%$. This variability provides a robust foundation for analyzing FM patterns and developing classification models.

### FM classification

The task of recognizing FM was translated into a binary classification problem between FM present (FM+) and FM absent (FM-). Various ML models were trained in three different configurations, using either the IMU, the RGB-D or both modalities simultaneously. Three different ML approaches were tested. The first that we refer to as Hand-Crafted Features (HCF) approach manually extracts simple statistical descriptors from each data channel in both time and frequency domains. The concatenated hand-crafted features are then used to train a Random Forest classifier. The second is based on learning features using a Multi-Branch Convolutional Neural Network (MBCNN). Finally, the last approach - that we refer to as Cross-Subject Adversarial Disentanglement (CSAD) - uses the MBCNN as backbone of a slightly modified feature disentanglement technique taken from Qian et al.^21^ to produce features that encode the concepts of subject identity and movement in an independent manner.

#### Hand-crafted features

As a baseline, we use a traditional feature engineering approach that consists in computing simple statistical descriptors from each sensor channel independently, followed by using the concatenated feature representation to train a Random Forest classifier for the binary classification of FM+ against FM-. A total of 29 features from both time and frequency domains described in Table 1 are computed separately from each channel. The computed feature vectors are concatenated channel-wise and used to represent each data frame. The dataset of features is then passed to a Random Forest classifier trained to recognize FM.

**Table 1:** List of the hand-crafted features computed channel-wise on both time and frequency domains.

Time domain	Frequency domain
Maximum, Minimum, Average, Standard-deviation,	Spectral energy
Percentile 20, Percentile 50, Percentile 80, Inter-quartile range,	Spectral entropy
Skewness, Kurtosis, Zero crossing, Auto correlation,	Spectral centroid
Mean of first order differences, L2 norm of first order differences,	Spectral rolloff
Mean of second order differences, L2 norm of second order differences,	Spectral flatness
Root Mean Square, Energy, Mean of signal jerk, Max of signal jerk,	High frequency energy ratio
L2 norm of signal jerk, Sample entropy, Permutation entropy	

#### Multi-Branch CNN

**Fig. 3:**
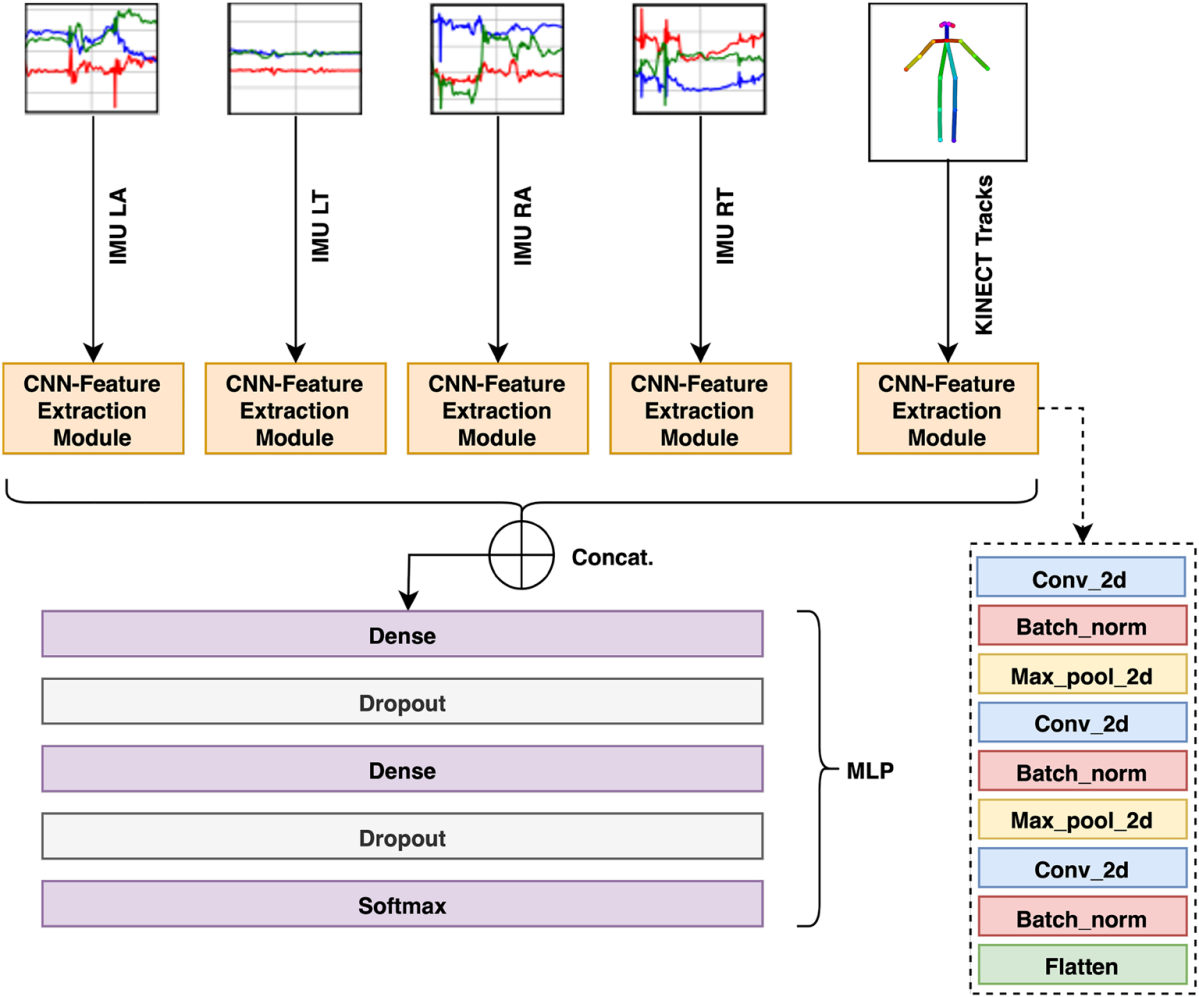
Multi-branch Convolutional Neural Network architecture for FM detection. LA, LT, RA and RT respectively refer to “Left Arm”, “Left Thigh”, “Right Arm” and “Right Thigh”.

We propose to use a relatively commonly used architecture in multivariate time series processing^22–24^ that consists in a MBCNN as baseline model, as illustrated in Fig. 3. The multivariate IMU time series data are sent to a four parallel convolutional branches, each processing the data from one IMU. The same is applied to the multivariate time series obtained by applying *DepthPose* to the RGB-D data stream, thus leading to a MBCNN with respectively four, one or five convolutional branches depending on whether the IMU, RGB-D or both modalities are used.

The features from all branches are concatenated and passed to a Multi-Layer-Perceptron (MLP) for classification. The MLP processes the concatenated features via two dense layers employing *l*2 regularization on the weights with dropout, followed by a final dense classification layer with softmax activation for class probability estimation. The model is trained using the cross-entropy loss.

#### Cross-subject adversarial disentanglement

**Fig. 4:**
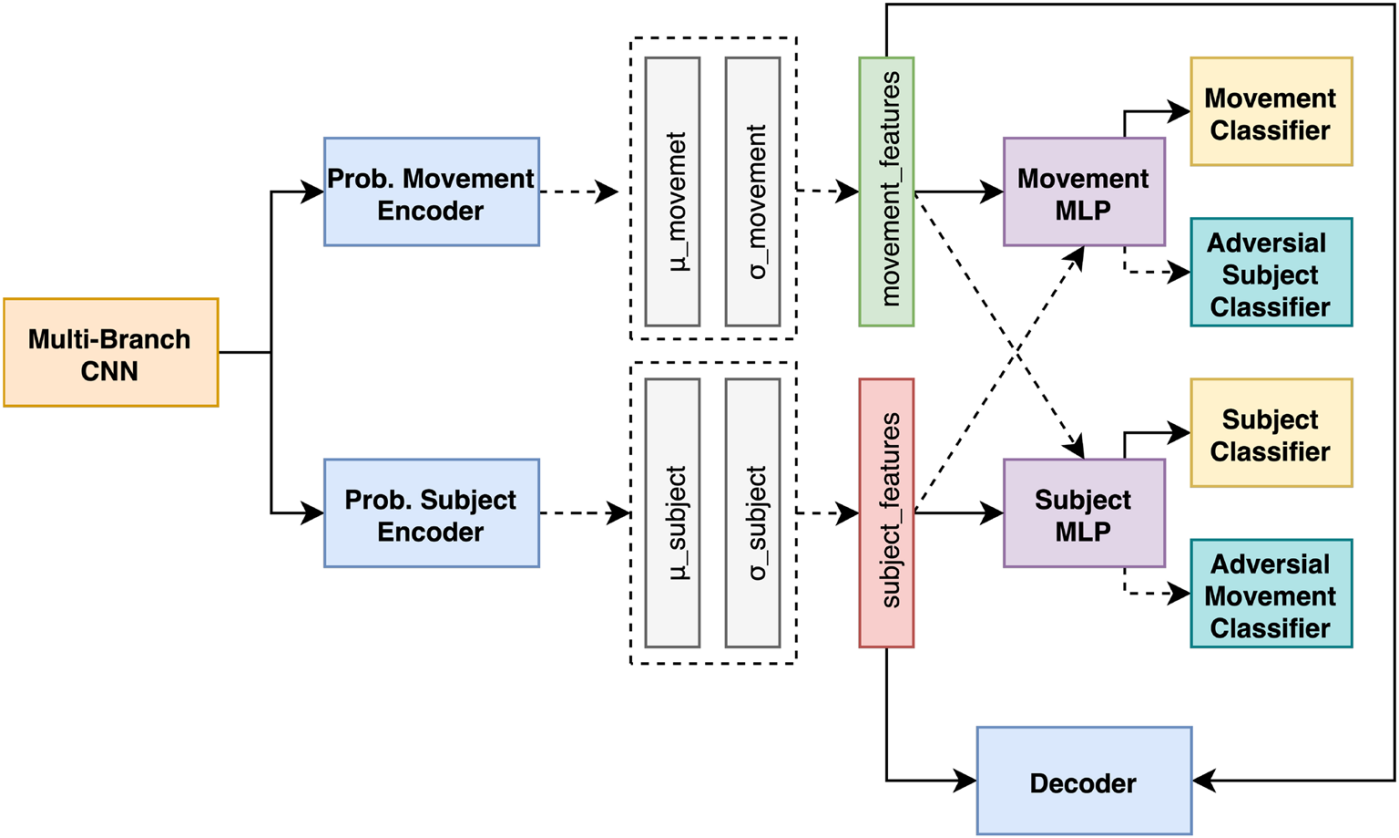
CSAD architecture for FM detection.

To increase the generalization capacity of the learned features, we also apply a feature disentanglement technique inspired by Qian et al.^21^ to produce features encoding movement independently from subject identity. As illustrated in Fig. 4, the CSAD is based on three main modules: a backbone feature extractor, an autoencoder component to obtain a disentangled latent feature representation, and an adversarial classification component to fine-tune the movement and subject features while ensuring their independence from one another.

We use the MBCNN introduced in the previous section as backbone model for CSAD, and send its features to two distinct $\beta$-Variational Autoencoder (VAE)^25^ trained by minimizing a reconstruction loss and the Kullback-Leibler (KL) divergence between the standard normal and learned latent space distributions. We refer to the outputs of the movement and subject VAE encoders as $z_m$ and $z_s$, respectively. To ensure that motion and subject-dependent patterns remain distinct, we perform a slight modification compared to the approach originally proposed by Qian et al.^21^ by using an adversarial module composed of a movement and subject MLP, and four classification MLPs. The movement MLP processes $z_m$ output through hidden layers and a softmax classifier trained with a Binary Crossentropy Loss (BCE) loss to yield a binary classification for FM. In its adversarial role, the same movement MLP processes $z_s$ through a second adversarial classifier trained with a negative Categorical Crossentropy Loss (CCE) loss to ensure that subject-related information is absent from the feature representation of the movement MLP. Conversely, the subject MLP processes $z_s$ through a subject classifier, and $z_m$ through an adversarial movement classifier to eliminate movement information from its feature representation. Finally, a decoder within the Dual-VAE framework reconstructs the original composite feature vector from the latent representations of both encoders. This reconstruction acts as a regularizer, ensuring that essential details of the original data are preserved, with its quality measured via the Root Mean Squared Error (RMSE) loss.

### Evaluation metrics

#### FM classification evaluation

We trained our models using a subject-independent stratified 5-fold cross validation. We split the 95 subjects across five folds so that the infants evaluated at risk by the clinical experts were evenly distributed between all folds, and that the data of each infant was assigned to only one fold. Models were then trained five times, each using one fold as testing set and the others as training set. This evaluation framework ensures a more realistic assessment of the classifier performances in a real-life application scenario where the test subject has not been seen during training. To evaluate the performance of FM classification, we used the accuracy, average F1-score (AF1), recall and precision averaged over all folds as evaluation metrics.

#### Evaluation of disentanglement

To evaluate the disentanglement of features with respect to the concepts of movement and subject identity, two different evaluation criteria were used: the first is a qualitative assessment based on the visualization of the learned features with T-distributed stochastic neighbor embedding (t-SNE) plots. For this evaluation, t-SNE plots were created for the train and test data sets of each fold, and samples were labeled once with subject and once with movement labels. The plots were analyzed to check the distribution of samples with respect to movement labels, and detect the presence of “subject clusters” in the feature space, which may indicate that the model takes decisions based on subjective information.

To supplement the visual evaluation, we introduce a second more quantitative criteria consisting in the Average Neighborhood Entropy (ANE) metric. The latter evaluates the distribution of subject labels in the feature space by averaging the local neighborhood entropy of the subject labels over all samples of the dataset. Let $(x_i)_{1 \le i \le N}$ be a dataset associated with labels $(y_i)_{1 \le i \le N}$ for the concept for which disentanglement is to be measured. The ANE parametrized by a neighborhood parameter $k \in \mathbb {N}$ is defined as follows:

1. **Identifying ***k*** nearest neighbors for each sample:** For each data sample $x_i$, the set $L_k(x_i)$ of *k* nearest neighbors according to a distance measure *d* is identified.
2. **Calculating Shannon entropy for each sample’s neighborhood:** The Shannon entropy of the label distribution within the neighborhood of each sample $x_i$, denoted as $H_k(x_i)$, is given by:
  1 $$\begin{aligned} H_k(x_i) = -\sum _{l \in \text {Unique}\{y_j | x_j \in L_k(x_i)\}} p(x_i,l) \log _2(p(x_i,l)) \end{aligned}$$
  where $p(x_i,l) = \frac{|\{x_j \in L_k(x_i) | y_j=l\}|}{|L_k(x_i))|}$ is the proportion of samples with label *l* within the neighborhood of $x_i$.
3. **Averaging all samples entropies:** The ANE is defined as the average of the entropy values $H(x_i)$ across all *N* samples in the dataset:
  2 $$\begin{aligned} \text {ANE}_k = \frac{1}{N} \sum _{i=1}^{N} H_k(x_i) . \end{aligned}$$

The ANE is used to assess how the samples of each subject are distributed in the feature space. A lower value for the ANE suggests the presence of subject clusters, while higher values indicate that the samples of each subject are more spread out in the feature space.

## Experiments and results

The results of the carried out experiments are presented in the following section for the HCF, MBCNN and CSAD approaches. Evaluations regarding both classification performances for the recognition of FM and feature disentanglement are provided. The reported classification performances are the evaluation metrics averaged over five repetitions of the model training and evaluation process. All experiments were ran using Tensorflow 2.7 on a machine equipped with an AMD Ryzen 9 5950X CPU and a NVIDIA GeForce RTX 3080 Ti GPU.

### HCF results

A total of 696, 2871 and 3567 features were respectively computed from all sensor channels in the configurations using the IMU data, the Kinect data and both simultaneously. The classification was performed by a Random Forest classifier whose optimal parameters were determined by random search on a manually defined parameter space, and are provided in the Appendix, section “Model Hyperparameters”.

#### FM-classification results

The evaluation metrics obtained in the configuration using Kinect data only, IMU data only and both modalities are shown in Tables 2, 3 and 4, respectively. In all configurations, the models managed to achieve high training performances. However, both the accuracy and the AF1 on the test dataset were notably lower, indicating that the features learned during training do not generalize to the unseen subjects of the test set. The best performances were obtained by the model trained using the IMU modality only, which yields a test AF1 of $54.60\%$ averaged over all folds.

**Table 2:** FM classification results obtained by the HCF approach using Kinect data.

Fold ID	Train	**Test**			
	Accuracy	Accuracy	F1-Score	Sensitivity	Specificity
1	97.14	58.68	53.24	66.47	40.69
2	97.52	52.56	44.59	57.73	33.83
3	97.14	55.28	50.88	57.23	49.58
4	96.96	61.77	53.50	72.27	34.89
5	97.01	58.46	55.60	61.58	51.81
**Average**	**97.15** $\varvec{\pm }$ **0.22**	**57.35** $\varvec{\pm }$ **3.53**	**51.56** $\varvec{\pm }$ **4.24**	**63.06** $\varvec{\pm }$ **6.34**	**42.16** $\varvec{\pm }$ **8.26**

**Table 3:** FM classification results obtained by the HCF approach using IMU data.

Fold ID	Train	Test			
	Accuracy	Accuracy	F1-Score	Sensitivity	Specificity
1	98.41	59.88	55.50	67.99	43.34
2	98.57	60.45	51.88	72.04	31.74
3	98.81	58.20	54.16	63.25	46.71
4	97.00	63.03	53.71	76.77	30.55
5	96.32	60.49	57.76	64.29	52.85
**Average**	**97.82** $\varvec{\pm }$ **1.10**	**60.41** $\varvec{\pm }$ **1.73**	**54.60** $\varvec{\pm }$ **2.19**	**68.87** $\varvec{\pm }$ **5.61**	**41.04** $\varvec{\pm }$ **9.66**

**Table 4:** FM classification results obtained by the HCF approach using both Kinect and IMU data.

Fold ID	Train	Test			
	Accuracy	Accuracy	F1-Score	Sensitivity	Specificity
1	96.97	58.41	53.35	65.88	41.61
2	97.49	52.54	44.81	57.72	34.18
3	97.30	55.25	50.38	57.57	48.20
4	96.97	62.44	54.15	72.85	35.66
5	96.85	58.95	55.69	62.80	50.58
**Average**	**97.11** $\varvec{\pm }$ **0.27**	**57.52** $\varvec{\pm }$ **3.77**	**51.67** $\varvec{\pm }$ **4.30**	**63.37** $\varvec{\pm }$ **6.37**	**42.05** $\varvec{\pm }$ **7.31**

#### Disentanglement results

**Fig. 5:**
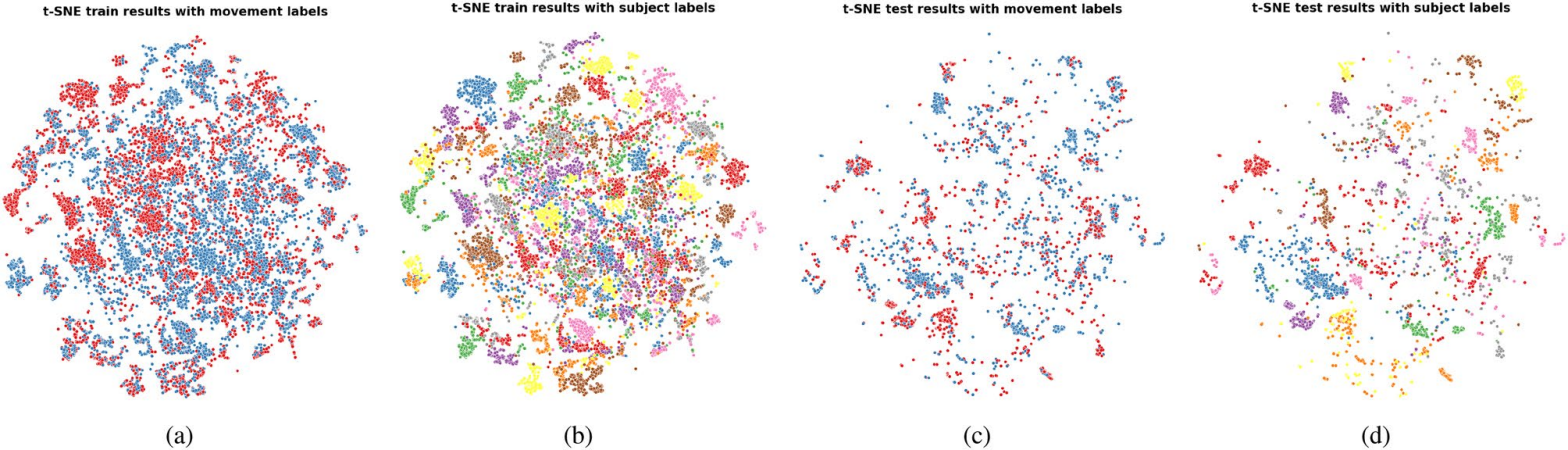
t-SNE plots representing the HCF feature space computed from the Kinect data. (**a**) Train set features annotated with movement labels; (**b**) Train set features annotated with subject labels; (**c**) Test set features annotated with movement labels; (**d**) Test set features annotated with subject labels.

**Fig. 6:**
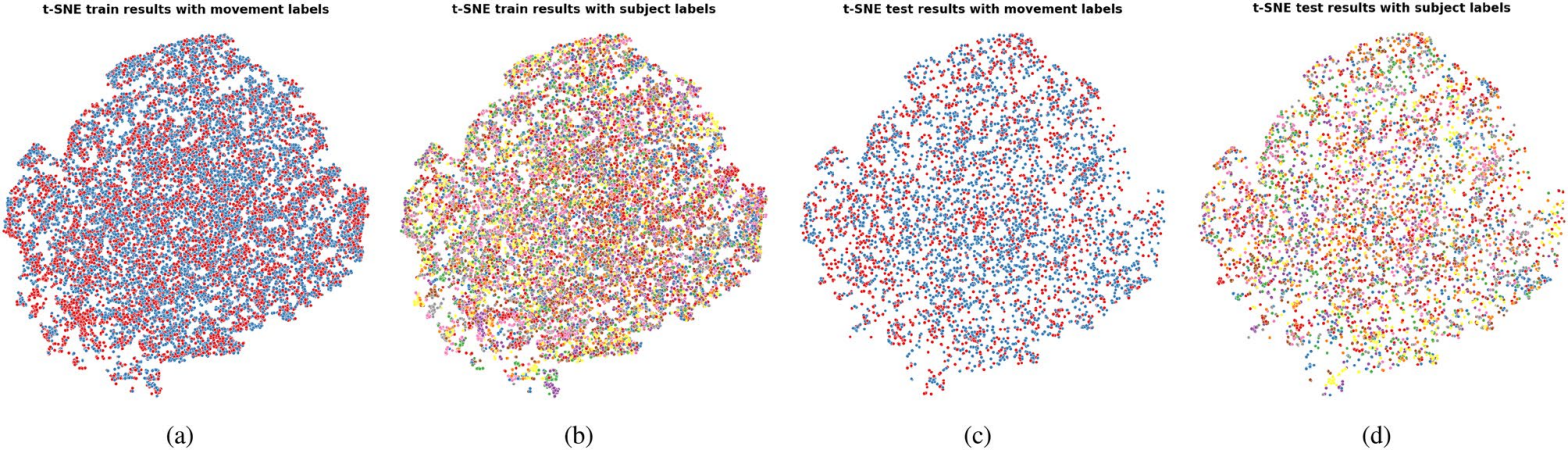
t-SNE plots representing the HCF feature space computed from the IMU data. (**a**) Train set features annotated with movement labels; (**b**) Train set features annotated with subject labels; (**c**) Test set features annotated with movement labels; (**d**) Test set features annotated with subject labels.

**Fig. 7:**
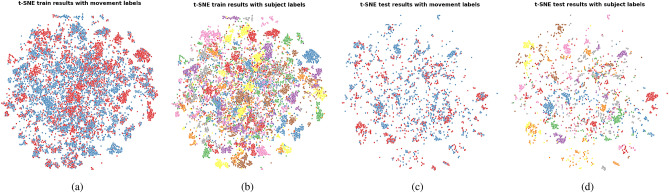
t-SNE plots representing the HCF feature space computed from both the Kinect and IMU data. (**a**) Train set features annotated with movement labels; (**b**) Train set features annotated with subject labels; (**c**) Test set features annotated with movement labels; (**d**) Test set features annotated with subject labels.

t-SNE plots showed that similar features were learned across folds in all tested configurations. Due to space limitations, we therefore show only the features learned on the first fold of each configuration. The t-SNE plots for the features computed on Kinect data, IMU data and both Kinect and IMU data are shown in Fig. 5, 6, and 7, respectively. The associated ANE scores computed for an arbitrarily chosen value of $k=10$ are provided in Table 5.

**Table 5:** ANE values (train | test) calculated with $k=10$ in the three tested configurations with HCF.

Fold	IMU	KINECT	IMU & KINECT
1	2.6313 | 2.2365	0.4894 | 0.2866	0.5587 | 0.3625
2	2.6665 | 2.0303	0.5085 | 0.2692	0.5850 | 0.3087
3	2.6551 | 2.0438	0.5458 | 0.1894	0.6261 | 0.2147
4	2.6575 | 2.1264	0.5097 | 0.2875	0.5867 | 0.3342
5	2.6775 | 1.9717	0.5226 | 0.2420	0.5930 | 0.2849
Average	2.6576 | 2.0817	0.5152 | 0.2549	0.5899 | 0.3010

The t-SNE plots show the presence of subject clusters when the features computed on the Kinect data are used for classification, and their apparent absence in the case where only the IMU data is used. The ANE scores confirm this observation as seen by their significantly lower values obtained in the two configurations involving Kinect features compared to the one with IMU features only. The lower FM classification performances obtained by the two models using Kinect features indicate that the presence of subject clusters in the training set is detrimental to the generalization capacity of the model to the unseen subjects of the test set.

### MBCNN results

To determine the MBCNN architecture leading to the best results, a randomized hyperparameter search was conducted on a manually defined parameter space. Details regarding the optimal hyperparameters are provided in the Appendix, section “Model Hyperparameters”. During the training of our models, we observed that the best test performances were often obtained at the very first epoch, at a point where models were still performing poorly on the training set, indicating a poor generalization capacity of the model across subjects of the dataset. The best epoch was therefore chosen by maximizing the mean accuracy across both the training and testing datasets. The network was trained using the Adam optimizer with a learning rate of $1e-4$, for 100 epochs with a batch size of 64.

#### FM-classification results

The model performances obtained in the configurations using Kinect data only, IMU data only and both modalities are shown in Tables 6, 7 and 8, respectively. Similarly to the HCF baseline, the features learned during the training phase do not generalize to the test set, as evidenced by the poor test metrics. The best performances were obtained by the model trained using the combination of both IMU and RGB-D modalities, which yields a test AF1 of $57.24\%$ averaged over all folds.

**Table 6:** FM classification results obtained by the MBCNN approach using Kinect data.

Fold ID	Train	Test			
	Accuracy	Accuracy	F1-Score	Sensitivity	Specificity
1	99.43	56.72	53.46	69.59	38.71
2	99.44	56.84	53.63	70.12	39.24
3	99.14	57.48	54.87	69.03	42.06
4	99.43	57.14	54.09	69.69	39.91
5	99.30	56.93	53.64	69.56	39.45
**Average**	**99.35** $\varvec{\pm }$ **0.13**	**57.02** $\varvec{\pm }$ **0.30**	**53.94** $\varvec{\pm }$ **0.54**	**69.60** $\varvec{\pm }$ **0.42**	**39.87** $\varvec{\pm }$ **1.22**

**Table 7:** FM Classification results obtained by the MBCNN approach using IMU data.

Fold ID	Train	Test			
	Accuracy	Accuracy	F1-Score	Sensitivity	Specificity
1	99.66	56.54	54.11	67.68	41.41
2	99.35	57.39	54.80	69.07	41.38
3	99.52	56.99	54.61	67.59	42.21
4	99.40	57.33	54.99	68.85	42.11
5	99.46	56.91	54.53	67.93	41.78
**Average**	**99.48** $\varvec{\pm }$ **0.12**	**57.03** $\varvec{\pm }$ **0.35**	**54.61** $\varvec{\pm }$ **0.32**	**68.22** $\varvec{\pm }$ **0.60**	**41.78** $\varvec{\pm }$ **0.33**

**Table 8:** FM Classification results obtained by the MBCNN approach using both IMU and Kinect data.

Fold ID	Train	Test			
	Accuracy	Accuracy	F1-Score	Sensitivity	Specificity
1	99.25	60.08	57.67	71.55	44.80
2	99.00	59.83	57.53	69.80	45.88
3	99.48	59.64	57.19	70.46	44.68
4	99.45	59.32	56.89	69.86	44.96
5	99.28	59.32	56.93	69.49	45.02
**Average**	**99.29** $\varvec{\pm }$ **0.20**	**59.64** $\varvec{\pm }$ **0.32**	**57.24** $\varvec{\pm }$ **0.33**	**70.23** $\varvec{\pm }$ **0.79**	**45.07** $\varvec{\pm }$ **0.44**

#### Disentanglement results

**Fig. 8:**
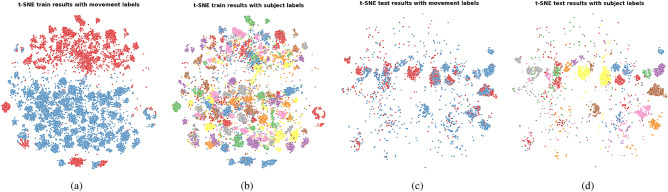
t-SNE plots representing the feature space learned by the MBCNN. (**a**) Train set features annotated with movement labels; (**b**) Train set features annotated with subject labels; (**c**) Test set features annotated with movement labels; (**d**) Test set features annotated with subject labels.

The t-SNE plots of the features learned by the MBCNN on the first fold using both Kinect and IMU data are shown in Fig. 8. Since the plots of the feature space computed on either IMU or Kinect data looked similar, we placed them in the Appendix, section “t-SNE plots”. The associated ANE scores are shown in Table 9 with $k=10$. The obtained scores are again relatively low, confirming the presence of subject clusters in the feature space observed in Fig. 8.

**Table 9:** ANE values (train | test) calculated with $k=10$ in the three tested configurations with MBCNN.

Fold	IMU	KINECT	IMU & KINECT
1	0.9024 | 0.7744	0.7292 | 0.4820	0.6119 | 0.4578
2	0.9437 | 0.5597	0.6034 | 0.3428	0.4002 | 0.2160
3	0.8477 | 0.5382	0.7543 | 0.4525	0.6333 | 0.3941
4	0.9528 | 0.7406	0.5630 | 0.3553	0.5639 | 0.4451
5	0.7879 | 0.3894	0.6867 | 0.5701	0.5594 | 0.4038
Average	0.8869 | 0.6005	0.6673 | 0.4405	0.5537 | 0.3834

### CSAD results

The backbone of the CSAD approach reused the optimal MBCNN architecture determined by the random parameter search described in the previous section. The network itself was trained using the Adam optimizer with a learning rate of $1e-4$, for 100 epochs. The parameters chosen for the Encoder - Decoder as well as then classification MLP are presented in the Appendix, section “Model Hyperparameters”.

#### FM classification results

Tables 10, 11, and 12 respectively present the results of the movement classification task in the three configurations using Kinect only, IMU only, and both combined. Similarly to the two previous approaches, the models managed to achieve high training performances, but significantly lower testing metrics, indicating that the features learned during training do not generalize to the unseen subjects of the test set. The best performances were obtained by the model trained using the IMU modality, which yields an AF1 of $53.02\%$ averaged over all folds.

**Table 10:** FM classification results obtained by the CSAD approach with Kinect data.

Fold_Id	Train		Test (Movement)			
	Accuracy-Movement	Accuracy-Subject	Accuracy	F1-Score	Sensitivity	Specificity
1	97.83	92.51	54.56	52.05	67.56	38.18
2	97.08	92.59	55.40	52.54	67.61	38.89
3	96.90	92.57	55.04	52.70	66.33	40.42
4	98.04	92.56	54.90	52.26	66.55	39.47
5	97.98	92.56	54.63	52.12	65.68	39.93
**Average**	**97.57** $\varvec{\pm }$ **0.48**	**92.56** $\varvec{\pm }$ **0.03**	**54.91** $\varvec{\pm }$ **0.32**	**52.33** $\varvec{\pm }$ **0.25**	**66.75** $\varvec{\pm }$ **0.74**	**39.38** $\varvec{\pm }$ **0.82**

**Table 11:** FM classification results obtained by the CSAD approach with IMU data.

Fold_Id	Train		Test (Movement)			
	Accuracy-Movement	Accuracy-Subject	Accuracy	F1-Score	Sensitivity	Specificity
1	94.78	90.98	54.82	53.04	64.98	42.67
2	93.04	91.02	55.71	53.36	67.33	40.44
3	94.54	90.98	54.93	52.91	65.49	41.36
4	94.81	90.98	55.28	53.38	65.01	43.02
5	95.62	90.99	54.83	52.41	66.65	39.42
**Average**	**94.56** $\varvec{\pm }$ ** 0.97**	**90.99** $\varvec{\pm }$ ** 0.02**	**55.11** $\varvec{\pm }$ ** 0.40**	**53.02** $\varvec{\pm }$ ** 0.38**	**65.89** $\varvec{\pm }$ ** 0.93**	**41.38** $\varvec{\pm }$ ** 1.22**

**Table 12:** FM classification results obtained by the CSAD approach with Kinect and IMU data.

Fold_Id	Train		Test (Movement)			
	Accuracy-Movement	Accuracy-Subject	Accuracy	F1-Score	Sensitivity	Specificity
1	85.11	82.12	58.20	52.62	74.89	34.24
2	86.70	82.16	57.75	52.09	76.52	31.60
3	85.40	82.13	58.95	53.37	74.73	35.09
4	87.32	82.22	58.35	53.81	72.17	37.70
5	86.87	82.14	57.69	52.08	75.03	33.09
**Average**	**86.28**$\varvec{\pm }$ **0.86**	**82.15** $\varvec{\pm }$ **0.04**	**58.19** $\varvec{\pm }$ **0.47**	**52.79** $\varvec{\pm }$ **0.69**	**74.67** $\varvec{\pm }$ **1.52**	**34.34** $\varvec{\pm }$ **2.12**

#### Disentanglement results

**Fig. 9:**
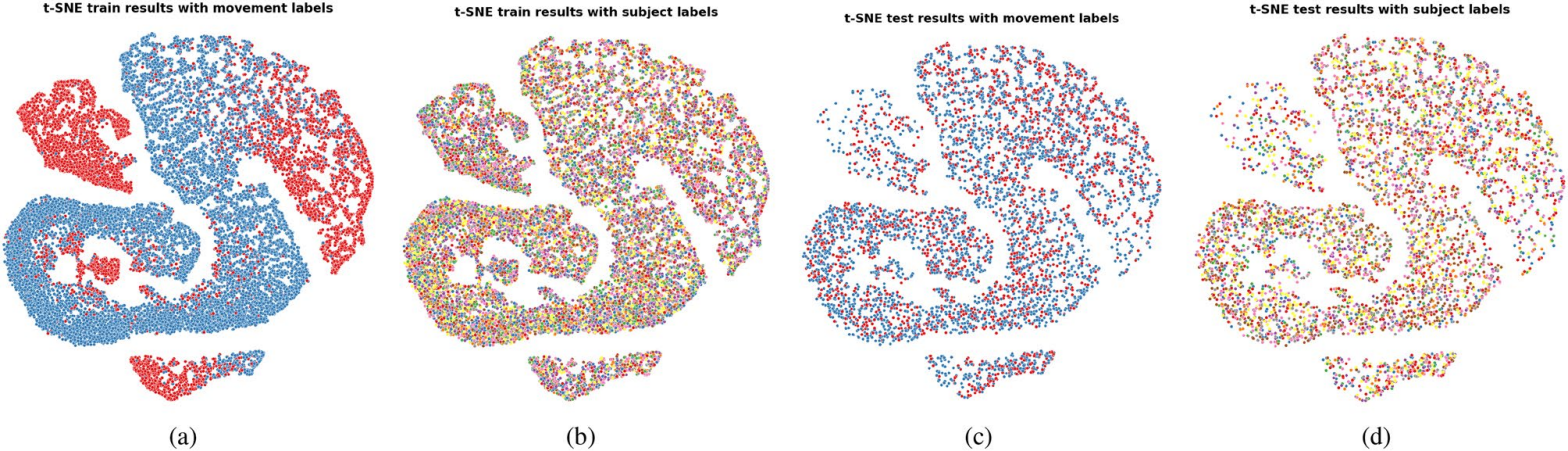
t-SNE plots representing the feature space learned by the CSAD approach. (**a**) Train set features annotated with movement labels; (**b**) Train set features annotated with subject labels; (**c**) Test set features annotated with movement labels; (**d**) Test set features annotated with subject labels.

Figure 9 shows the t-SNE plots of the features learned on the first fold by CSAD using IMU data. Due to their similarity, the plots for the configurations Kinect only and both IMU and Kinect are placed in the Appendix, section “t-SNE plots”. Compared to the two previous approaches, a clear improvement can be observed in the disentanglement of subject and movement features, as the t-SNE plots of both train and test sets no longer show any subject clusters. While the train data still shows clear clusters for movement, the same does not apply to the test data, which is reflected by the poor test classification performances. The large associated ANE scores reported in Table 13 confirm the lack of observed subject clusters in the feature space.

**Table 13:** ANE values (train | test) calculated with $k=10$ in the three tested configurations with CSAD.

Fold	IMU	KINECT	IMU & KINECT
1	3.133 | 2.8273	3.1222 | 2.7742	3.1197 | 2.7132
2	3.1370 | 2.6635	3.1361 | 2.6686	3.1300 | 2.7815
3	3.1374 | 2.7545	3.1403 | 2.7025	3.1410 | 2.7694
4	3.1419 | 2.7508	3.143 | 2.7398	3.1276 | 2.7374
5	3.1306 | 2.8269	3.124 | 2.7348	3.1247 | 2.7225
Average	3.1360 | 2.7646	3.1331 | 2.7240	3.1286 | 2.7448

### Comparative results

We compare the performances of the HCF, MBCNN and CSAD approaches by applying a Friedmann test with Nemenyi post-hoc. The test is carried out once for each evaluation metric, using the sets of metrics obtained in the five repeated runs for each approach. To account for the multiple comparisons, a Bonferroni correction is applied to the significance level of 0.05, leading to a corrected significance level of $\alpha =0.0056$. Table 14 shows a comparison of the metrics obtained by the three tested approaches in the three sensor input modality configurations (Kinect, IMU, and both simultaneously). The results show that for all metrics, most approaches yield performances that are statistically equivalent. More specifically, we use the test F1-score as main evaluation metric, since it is the least affected by biases induced by class imbalance. From its observation, we can conclude that all methods are statistically equivalent to the best performing one - MBCNN using IMU and Kinect data - with the exception of HCF with Kinect, and HCF with both IMU and Kinect.

**Table 14:** Comparative performances for FM classification obtained by the HCF, MBCNN and CSAD, averaged across five repeated runs. The best performances for each metric are displayed in bold font. * indicates that the metric is statistically different from the best one according to metric-wise post-hoc Nemenyi tests using a significance level with Bonferroni correction of $\alpha =0.0056$.

Approach	Modality	Train	Test			
		Accuracy	Accuracy	F1-Score	Sensitivity	Specificity
HCF	Kinect	97.15 ± 0.23	57.35 ± 0.19	51.56 ± 0.15*	63.06 ± 0.30*	42.16 ± 0.35
	IMU	97.82 ± 0.25	**60.41** $\varvec{\pm }$ **0.26**	54.60 ± 0.25	68.87 ± 0.33	41.04 ± 0.52
	IMU & Kinect	97.11 ± 0.06	57.52 ± 0.35	51.67 ± 0.47*	63.37 ± 0.63*	42.05 ± 1.20
MBCNN	Kinect	99.35 ± 0.13	57.02 ± 0.30	53.94 ± 0.57	69.60 ± 0.39	39.87 ± 1.29
	IMU	**99.48** $\varvec{\pm }$ **0.12**	57.03 ± 0.34	54.61 ± 0.33	68.22 ± 0.69	41.78 ± 0.39
	IMU & Kinect	99.29 ± 0.19	59.64 ± 0.33	**57.24** $\varvec{\pm }$ **0.35**	70.23 ± 0.82	**45.07** $\varvec{\pm }$ **0.47**
CSAD	Kinect	97.56 ± 0.54	54.91 ± 0.34*	52.33 ± 0.28	66.75 ± 0.83	39.38 ± 0.88*
	IMU	94.56 ± 0.94	55.11 ± 0.38*	53.02 ± 0.40	65.89 ± 1.05	41.38 ± 1.51
	IMU & Kinect	86.28 ± 0.97*	58.19 ± 0.51	52.79 ± 0.77	**74.67** $\varvec{\pm }$ **1.57**	34.34 ± 2.29*

## Discussion

### Study results

The classification performances shown in the previous section indicate the difficulty to obtain features for the classification of FM that properly generalize across subjects. Both IMU and RGB-D modalities fail to lead to robust features, with the best performances being obtained by the MBCNN trained using both inertial and video modalities (AF1 of $57.24 \pm 0.33\%$). It should be noted that despite returning the highest evaluation metrics, a visual inspection of its features showed that the MBCNN likely incorporated subjective information learned from the training subjects in its decision process. This is an indication of shortcut learning^26^ likely happening, that refers to the model learning information irrelevant to the target problem to minimize its training loss, which leads to poor generalization capabilities. This phenomenon is quite common in studies that involve the collection of data with potentially large inter-subject variability, which concerns both visual and IMU modalities^18,27^, and is not detected by traditional classification metrics. While the CSAD approach failed to yield better classification performances in our study, we argue that without a training dataset that contains a very large number of diverse subjects representative of the general population, any model incorporating subjective information in its decision process will face difficulties to generalize to unseen subjects for the recognition of highly subjective patterns (such as FM).

The test evaluation metrics we obtained in our study seem to be in-line with the ones from the related literature reported in a subject-independent setup^10,11^. The only exception are provided by two studies from Kulvicius et al^.16,17^ involving the usage of pressure mats, either used alone or in addition to RGB and IMU modalities. While the classification performances reported in a subject-independent setup by both studies look very promising, it can be pointed out that the data of only 45 infants were used in both of them, which is below average compared to the other studies from the literature. Overall, it can be observed from past studies that the larger the number of subjects is, the lower classification performances tend to get. The studies with the largest sample size were carried out by Morais et al.^11^ who used two datasets of 1343 and 739 videos, but reported a relatively low AUROC of $75.12\%$/$69.37\%$, and a specificity at $75\%$ sensitivity of $60.16\%$/$56.20\%$ for a binary classification task on both respective datasets. This suggests that finding robust features generalizing across a large set of subjects becomes progressively more difficult as the diversity in the dataset increases.

The results of our study indicate that both video and inertial data are insufficient to obtain proper classification performances for the recognition of FM, either when used separately or combined together. We therefore carried out investigations to understand the shortcomings of both modalities. Our main assumption for the video modality is that the tracking accuracy of *OpenPose *- the most notable state-of-the-art tracking algorithm at the time of our study - is not fine-grained enough to accurately capture the subtle oscillations that characterize FM. Studies assessing its accuracy in the literature are uncommon, due to the difficulty to obtain some accurate ground truth against which the estimated coordinates of the tracked keypoints should be compared to. Nakano et al.^28^ carried out a study to evaluate the accuracy of *OpenPose* in tracking two subjects performing three motor tasks. They reported that *OpenPose* yields an error lower than 30*mm* $80\%$of the time, which while acceptable for many applications may be too large for the problem of detecting FM. Groos et al.^29^ reported that the infant-specific version of *OpenPose* proposed by Chambers et al.^7^ was insufficient for optimal pose estimation in one of their studies. These observations were tested on our dataset by performing a qualitative analysis consisting in asking one clinical expert who contributed to the data annotation process to visualize videos of fidgety infants, with the keypoints and skeletons detected by *DepthPose *superimposed on the videos. It was observed that the tracking was sometimes unstable during non-fidgety periods, and conversely relatively stable at times where the infants were fidgety. This lines up with recent observations from Gama et al.^30^ and Kulvicius et al.^17^ who both recommended to use *ViTPose*^31^ over *OpenPose* due to its increased stability.

We hypothesize that the low classification performances obtained when using the IMU data stem from problems regarding the standardization of the IMU placement and orientation across subjects. Detailed instructions regarding the IMU setup were provided to the clinicians who carried out the GMA on the infants, both in terms of position or orientation. But despite the care taken in standardizing the experimental protocol, setup inconsistencies may have occurred from one infant to another due to various practical considerations (e.g. uncooperative infants, time constraints incurred by the doctors’ busy schedule, different infants’ morphologies, etc.). This high variance in IMU placement could increase the difficulty of ML models learning features that are robust from one subject to another. Some past related studies have attempted to address this issue by using either body-suits with specific pockets for the IMU^17^, or straps to be attached around the infants’ limbs^12^ in their experimental protocols. But these remain sensitive to the high variance in infants’ morphology.

### Practical recommendations

To address the aforementioned obstacles to obtaining robust classification performances for the detection of FM using ML techniques, we issue the following practical recommendations to researchers interested in investigating the automation of GMA:

- Care should be taken regarding the selection of the body tracking algorithm in RGB videos, in particular regarding its accuracy due to how subtle FM are. Recent work by Kulvicius et al.^17^ for instance preferred to use *ViTPose*^31^ over the traditionally used *OpenPose*, reporting an improved accuracy of the former. It should also be considered that most body tracking algorithms are usually trained on video datasets containing mostly adults, which may lead to degraded performances when used on videos of infants. While a recent study by Gama et al.^30^ showed that several state-of-the-art body tracking algorithms - including *ViTPose* - could obtain competitive performances without fine-tuning for infant body tracking, domain adaptation of the tracking models using datasets of infant videos may be required to reach a level of performances that is precise enough for subtle movements such as FM. For this reason, the publication of additional FM-specialized heterogeneous infant datasets to train more accurate pose estimation models for infants, as done by Groos et al.^29^, would be beneficial to the research community.
- For studies involving the use of inertial sensors, we recommend to standardize their placement on the infants with the help of properly fitted body-suits such as the MAIJU jumpsuits used by Airaksinen et al.^32^. It can be noted that differences in infants’ morphologies can still lead to standardization issues. To mitigate this, devices equipped with either a gravimeter or magnetometer could be used to estimate the orientation of each IMU in a common world referential^33^, and use this information to obtain features that are independent from each specific IMU orientation^34^.
- Additional sensor modalities could be considered to provide complementary information to the traditionally used vision and inertial sensors. Promising performances leveraging pressure mats have in particular been reported in past related work^16,17^. These devices could be tested on additional cohorts in future studies to provide further insights on their performances in different clinical environments.
- Finally, we advise caution regarding the evaluation of ML models trained for the recognition of FM, since commonly used classification metrics may not detect shortcut learning. To ensure that robust features that generalize across subjects can be learned, we recommend to check for the disentanglement between the concepts of identity and movement. This can be performed by either visual confirmation, e.g. via t-SNE plots of the features, or by using more objective metrics such as the ANE introduced in this article.

### Limitations and future work

Despite the care taken to carry out this study, some limitations to its scope should be addressed in future work. Firstly, the CSAD approach tested in our study failed to yield improvements in FM classification performances compared to the other approaches, even though it managed to learn features that are independent from subject identity information. These underwhelming performances could be attributed to two different factors: either the target FM classification task also relies on other concepts not characterized by the acquired FM and subject labels, or the movement features learned by CSAD still contain “impurities”, i.e. information unrelated to movement that is detrimental to the target task^35^. Addressing either of these issues is not trivial without an a priori knowledge of the latent factors that could play a role in FM recognition. Nevertheless, future work could investigate the performances of feature disentanglement methods not part of the family of concept bottleneck models, such as post-hoc concept extraction approaches that would leverage powerful models pre-trained for activity recognition to learn relevant concepts for FM recognition^35^. Secondly, attempts to fine-tune other state-of-the-art body tracking algorithms such as *VitPose* on infant RGB video datasets will be carried out in future work in order to address the limitations related to insufficient precision observed with the current tracking algorithm that built upon *OpenPose*. Finally, the scope of the study could be expanded by applying the tested approaches on different datasets incorporating FM annotations, with further refined or additional architectures for deep feature learning.

## Conclusion

In this paper, a study aiming at automating the recognition of FM in the frame of GMA using machine learning techniques was investigated. Both RGB-D cameras and IMU were used to acquire data from a cohort of 95 infants aged 15 weeks or less. The data were annotated by clinical experts proficient in the recognition of FM, and used to train machine learning models for the binary classification problem of whether FM are present or absent. Convolutional-based architectures either used on their own or in conjunction with feature disentanglement techniques were applied using either the RGB-D, inertial or both modalities. Our results show that for all tested combinations of modalities, it remains very challenging to obtain a feature representation that properly generalizes to unseen subjects. More specifically, we identified limitations related to the precision of the body tracking algorithm that remains insufficient for the detection of movements as subtle as FM, and to the standardization in the placement of IMU across subjects. Our recommendations for future work in the direction of the automation of FM detection include the selection of more precise body tracking algorithms that underwent domain adaptation on video datasets of infants, the standardization of IMU placement using body suits, the usage of additional modalities (e.g. pressure mats) and the acquisition of data from additional cohorts to further test the generalization capacities of the developed systems.

## Supplementary Information

Supplementary Information.

## Data Availability

Due to a clause regarding data usage and privacy included in the ethics application submitted for this study (Ethics Commission of the University of Lübeck, Germany, Aktenzeichen 21-319), the RGB-D videos of the infants cannot be shared. Anonymized data such as the *DepthPose* tracks, IMU data and processed version of the annotations are available from the corresponding author on reasonable request.
