# Supplementary material for: A machine learning study highlighting the challenges of fidgety movement recognition using vision and inertial sensors
Source: Sci Rep. 2026 Jan 5;16:459. doi: 10.1038/s41598-025-28523-3 (PMC12775505; doi:10.1038/s41598-025-28523-3)
Supplement: Supplementary file 1 — Supplementary Information. [file 41598_2025_28523_MOESM1_ESM.pdf]

Appendix

Model hyperparameters

The best parameters of the Random Forest classifier used with the HCF and determined by a random search on a manually defined parameter space are provided in Supplementary Table 1.

Parameter	Value
Number estimators	600
Max depth	22
Decision threshold	0.60
Min sample leaf	1
Min sample split	2
Max samples	0.9
Bootstrap	True

Supplementary Table 1. Optimal parameters of the Random Forest classifier in the HCF approach

To determine the neural architecture leading to the best results for both MBCNN and CSAD, a randomized hyperparameter search was conducted. The space of parameters is defined in Supplementary Table 2.

Hyperparameter	Values
Batch Size	32, 64, 128
Epochs	25, 50, 100
Loss Function	sparse_categorical_crossentropy
Learning Rate	1e-1, 1e-2, 1e-3, 1e-4
Optimizer	Adam, Adamax, Adagrad, Adadelta
Regularizer CNN IMU	l1, l2, l1_l2, None
l1 Rate CNN IMU	1e-3, 1e-4
l2 Rate CNN IMU	1e-1, 1e-2, 1e-3, 1e-4
Regularizer CNN KINECT	l1, l2, l1_l2, None
l1 Rate CNN KINECT	1e-3, 1e-4
l2 Rate CNN KINECT	1e-1, 1e-2, 1e-3, 1e-4
Regularizer MLP	l1, l2, l1_l2, None
l1 Rate MLP	1e-3, 1e-4
l2 Rate MLP	1e-1, 1e-2, 1e-3, 1e-4
Activation CNN IMU	tanh, relu
Activation CNN KINECT	tanh, relu
Activation MLP	tanh, relu
CNN Feature maps IMU	[16, 32, 64], [64, 32, 16], [16, 16, 16], [32, 32, 32]
CNN Kernel sizes IMU	[10, 5, 3], [5, 5, 5], [3, 3, 3]
CNN Feature maps KINECT	[16, 32, 64], [64, 32, 16], [16, 16, 16], [32, 32, 32]
CNN Kernel sizes KINECT	[3, 3, 3]
MLP Neurons List	[256, 128], [128, 64]
Drop1 Rate	0.1, 0.2, 0.3, 0.4
Drop2 Rate	0.1, 0.2, 0.3, 0.4

Supplementary Table 2. Hyper-parameters and their values tested during random search hyper-parameter optimization by using the MBCNN

For the MBCNN, the best parameters for the convolutional block of the FM classifier are shown in Supplementary Tables 3 and 4 for the branches processing the IMU and RGB-D data, respectively. The best parameters of the MLP including type, regularization strength and dropout rates are shown in Supplementary Table 5.

Layer	#_Maps	Kernel	Activation
Conv_0	16	(10, 1)	TanH
Max_pool_0	X	(3, 1)	X
Conv_1	32	(5, 1)	TanH
Max_pool_1	X	(3, 1)	X
Conv_2	64	(3, 1)	TanH

Supplementary Table 3. Model parameters for the convolutional block processing the IMU data. #_maps refers to the number of feature maps per layer, "Kernel" to the dimensions of the convolution kernels, and "Activation" to the activation function.

Layer	#_Maps	Kernel	Activation
Conv_0	16	(3, 1)	ReLU
Max_pool_0	X	(2, 1)	X
Conv_1	16	(3, 1)	ReLU
Max_pool_1	X	(2, 1)	X
Conv_2	16	(3, 1)	ReLU

Supplementary Table 4. Model parameters for the convolutional block processing the Kinect data. #_maps refers to the number of feature maps per layer, "Kernel" to the dimensions of the convolution kernels, and "Activation" to the activation function.

Layer	Neurons / Dropout_Rate	Regulaizer(rate)	Activation
dense_0	256	L2(0.001)	TanH
dropout_0	0.3	X	X
dense_1	128	L2(0.001)	TanH
dropout_1	0.3	X	X
dense_classification	2	X	Softmax

Supplementary Table 5. Model parameters for the MLP

For the CSAD, the MBCNN previously defined whose hyperparameters were set via random search was used as backbone feature extractor. The parameters of the other components (VAE, movement and subject MLP) were determined empirically since few tested combinations ended up leading to convergence of the loss during training. All chosen hyperparameters are presented in Supplementary Tables 6, 7, and 8 for the VAE encoder, VAE decoder and MLP, respectively.

Layer	Neurons	Activation
dense_0	512	TanH
dense_1	256	TanH
dense_mean, dense_variance	128, 128	X
dense_sampling	128	X

Supplementary Table 6. CSAD VAE encoder hyperparameters

Layer	Neurons	Activation
dense_0	512	TanH
dense_1	8640	TanH

Supplementary Table 7. CSAD VAE decoder hyperparameters

Layer	Neurons / Dropout_rate	Activation
dense_0	128	TanH
dropout_0	0.1	X
dense_1	128, 128	TanH
dropout_1	0.1	X
dense_classification,<br>dense_adversial_classification	2 or 95,<br>95 or 2	Softmax

Supplementary Table 8. Model parameters of the Dual-MLP with four classifiers.

Additionally, the CSAD model was trained using the following loss term $\mathscr{L}$:

$$\mathscr{L} = L_r + \lambda_1 L_{KL,m} + \lambda_2 L_{KL,s} + \alpha_1 L_{cls,m} + \alpha_2 L_{cls,s} + \beta_1 L_{adv,m} + \beta_2 L_{adv,s} \quad (3)$$

where:

- L_r is the VAE reconstruction loss defined as the RMSE computed between the decoder output and MBCNN output.
- $L_{KL,m}$ and $L_{KL,s}$ refer to the KL divergences between the latent space distributions of the movement and subject VAE encoders respectively, and the standard normal distribution.

- $L_{cls,m}$ and $L_{cls,s}$ are the classification losses for movement and subject, chosen as binary and categorical cross-entropies, respectively.

- $L_{adv,m}$ and $L_{adv,s}$ are the adversarial losses for movement and subject classification, respectively chosen as the negative binary and categorical cross-entropies.

The weighting factors λ_1 and λ_2 were chosen to be small and both set to 0.2, as larger values led the network to no longer be able to classify movements and subjects, or reconstruct the input of the encoder. The weighting factors β_1 and β_2 for the adversarial classifiers also had to be chosen relatively low and were set to 0.1, since the adversarial errors quickly led to negative values with large absolute values, resulting in an unstable training. The weighting factors for the classification losses α_1 and α_2 were set to 1.0.

t-SNE plots

The following Supplementary Figures show the t-SNE visualization of the feature representations learned by the MBCNN and CSAD approach. Supplementary Figs. 1 and 2 respectively represent the features obtained by the MBCNN trained with the IMU and RGB-D *OpenPose* tracks. Supplementary Figs 3 and 4 show the features learned by the CSAD approach with the IMU and RGB-D *OpenPose* tracks, respectively. Due to space considerations and strong similarities between the learned feature space across different folds, only the plots obtained on the first fold are shown.

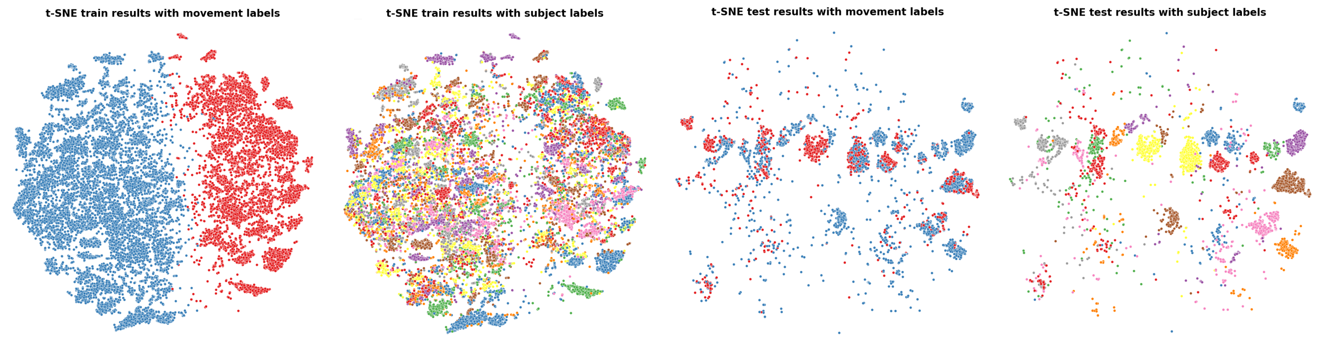

(a) (b) (c) (d)

Supplementary Figure 1. t-SNE plots representing the feature space learned by the MBCNN using IMU data. a) Train set features annotated with movement labels; b) Train set features annotated with subject labels; c) Test set features annotated with movement labels; d) Test set features annotated with subject labels.

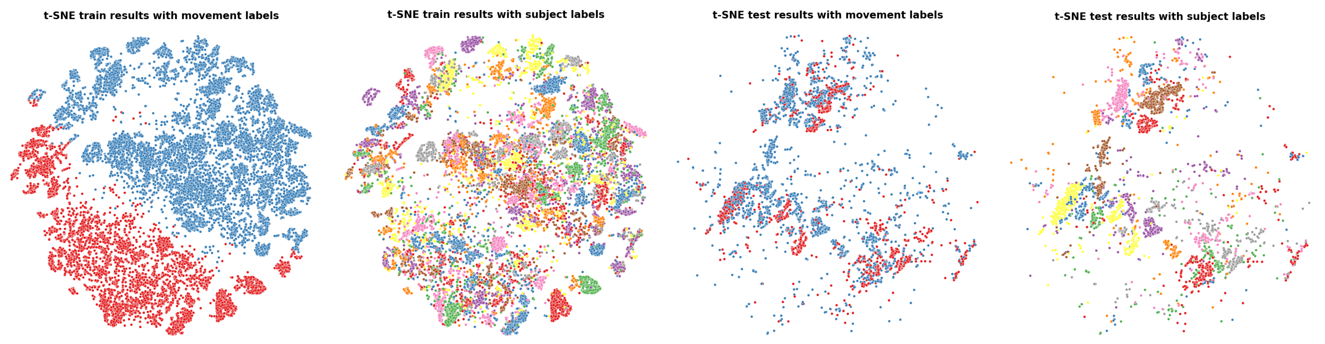

(a) (b) (c) (d)

Supplementary Figure 2. t-SNE plots representing the feature space learned by the MBCNN using the *OpenPose* tracks. a) Train set features annotated with movement labels; b) Train set features annotated with subject labels; c) Test set features annotated with movement labels; d) Test set features annotated with subject labels.

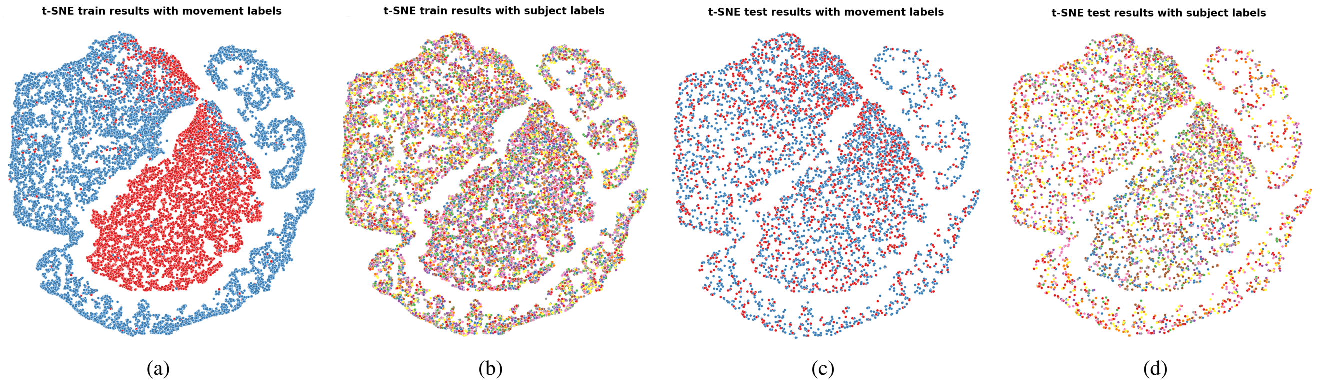

Supplementary Figure 3. t-SNE plots representing the feature space learned by the CSAD approach using IMU data. a) Train set features annotated with movement labels; b) Train set features annotated with subject labels; c) Test set features annotated with movement labels; d) Test set features annotated with subject labels.

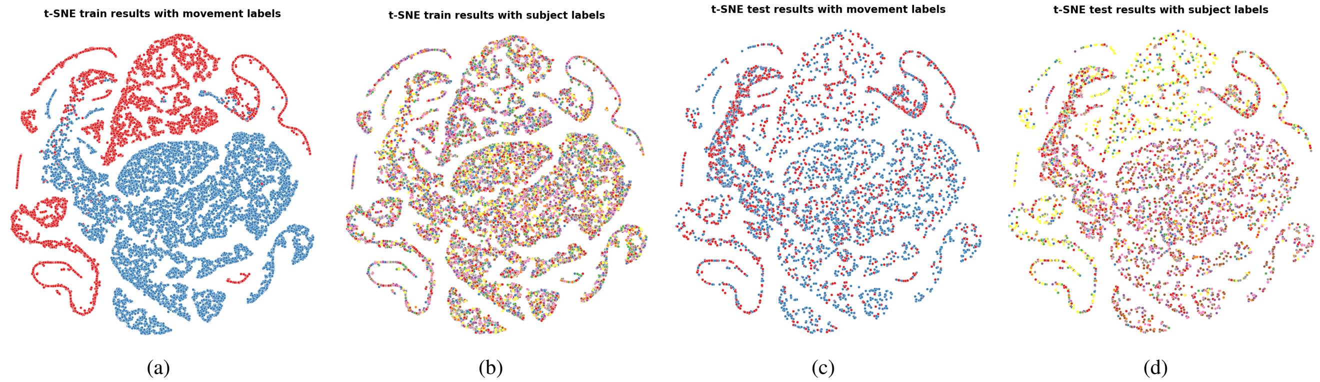

Supplementary Figure 4. t-SNE plots representing the feature space learned by the CSAD approach using the *OpenPose* tracks. a) Train set features annotated with movement labels; b) Train set features annotated with subject labels; c) Test set features annotated with movement labels; d) Test set features annotated with subject labels.
